# Re-evaluating our language when reducing risk of SARS-CoV-2 transmission to healthcare workers: Time to rethink the term, “aerosol-generating procedures”

**DOI:** 10.1186/s12985-022-01910-2

**Published:** 2022-11-17

**Authors:** Andrew Silvers, David J. Brewster, Alister Ford, Ana Licina, Cassandra Andrews, Mark Adams

**Affiliations:** 1 Department of Anaesthesia, Monash Health, Clayton, VIC 3168 Australia; 2 Central Clinical School, Faculty of Medicine, Monash University, Melbourne, VIC Australia; 3 Intensive Care Unit, Cabrini Hospital, Malvern, VIC Australia; 4 Royal Victorian Eye and Ear Hospital, Melbourne, VIC Australia; 5 Dandenong Hospital, Monash Health, Dandenong, VIC Australia; 6 Department of Anaesthesia and Perioperative Medicine, Monash Health, Melbourne, VIC Australia

**Keywords:** COVID-19, Transmission, Health care worker, Communicable diseases, Delivery of health care

## Abstract

The term, "aerosol-generating procedures” (AGPs), was proposed during the prior SARS-CoV-1 epidemic in order to maximise healthcare worker and patient protection. The concept of AGPs has since expanded to include routine therapeutic processes such as various modes of oxygen delivery and non-invasive ventilation modalities. Evidence gained during the SARS-CoV-2 pandemic has brought into question the concept of AGPs with regard to intubation, airway management, non-invasive ventilation and high flow nasal oxygen delivery. Although encounters where these procedures occur may still be associated with increased risk of infectious transmission, this is a function of the clinical context and not because the procedure itself is aerosol-generating.

## Introduction

At the start of the SARS-CoV-2 pandemic, approaches to minimising transmission risk from patients to healthcare workers (HCWs) in Australia had to be developed. This was driven by minimal data availability, an unvaccinated population, uncertain personal protective equipment (PPE) supply chains, limited access to fit testing and an understandable level of anxiety on the part of HCWs. To provide urgently needed direction, guidelines were developed based largely on extrapolation of data from the 2003 severe acute respiratory syndrome (SARS) epidemic and from the Middle East Respiratory syndrome coronavirus (MERS-CoV) [1–4]. It is now appropriate to re-evaluate best language when aiming for reduction of risk of transmission to HCWs in the context of SARS-CoV-2 pandemic with consideration of new evidence.

HCWs involved in these procedures are undoubtedly at increased risk of infection through prolonged close contact with an infected patient [5]. The mechanism of infection, however, is more likely from exposure to aerosols, droplets and fomites from the coughing dyspnoeic patient rather than from the AGP itself.

## Aerosol-generating procedures—definitions and origins

The early guidelines emphasized the importance of the “aerosol-generating procedure” (AGP); defined as any medical procedure that can induce the production of aerosols of various sizes, including small (< 5 µm) particles [6]. The concept of AGPs has been based largely on their association with disease transmission in HCWs during the SARS-1 epidemic in 2003. Expert consensus around which procedures constitute an AGP has not been achieved [7] Many organisations, including the Center for Disease Control and Prevention, currently list medical procedures which are often considered AGPs [7, 8] (see Table 1).

**Table 1:** Aerosol-generating procedures [7–9]

Aerosol-generating procedures
Bronchoscopy
Tracheal intubation
Non-invasive ventilation (for example, BiPAP, CPAP)
High-flow nasal oxygen therapy
Manual ventilation before intubation, intubation
Ventilation via supraglottic airways (including insertion and removal)
Cardiopulmonary resuscitation
Open airway suctioning
Tracheostomy
Sputum induction
Nebuliser use

These procedures were identified as potentially capable of generating particles less than 5 µm in diameter that could contain live virus, remain suspended in air for extended periods, penetrate deep into the alveolar tree, thereby increasing the risk of transmission and serious illness. This list of procedures has formed the base dataset for an ever-expanding list via extrapolation to other ‘like’ procedures, all of which subsequently required ‘aerosol precautions’. Similarly, procedures or management that did not constitute aerosol risk were managed with ‘droplet and contact precautions’ only.

## Consequences of focusing on AGPs during the SARS-CoV-2 pandemic

Early international experience with SARS-CoV-2 demonstrated the importance of reducing HCW infection, and AGPs quickly became a focus of concern. Operating room environments and critical care areas conduct many airway procedures. Contrary to expectations, infections in critical care staff were comparatively low [10].

In the operating room, routine airway management interventions were considered AGPs, including intubation, extubation, and laryngeal mask insertion [7–9]. The end-product of this universal approach to risk mitigation resulted in consumption of limited PPE reserves. Additional measures such as removal of medications and equipment from operating rooms occurred where AGPs took place. These created complexity and delays in delivering care, with the potential for patient harm. Operating room efficiency was impaired due to the need to accommodate aerosol-clearance times [11] and recover patients within the operating room. Further to this, there was a reluctance to initiate high flow humidified nasal oxygen therapy due to concerns about its potential to generate aerosols. Facemask ventilation and even external cardiac compressions were deemed AGPs and some hospital cardiac arrest protocols were altered to mandate donning of aerosol PPE before initiating cardiopulmonary resuscitation, delaying resuscitative care to our most critically unwell patients.

Epidemiological data from the SARS-CoV-1 epidemic shows an association between intubation of patients with SARS and disease transmission to HCWs [2]. Closer analysis of this data reveals that patient airway procedures tended to occur in the context of prolonged contact of multiple clinicians, likely within a confined space with a coughing, dyspnoeic, highly infective patient being intubated for respiratory failure [12]. Transmission rates increased with higher acute physiology and chronic health evaluation-2 (APACHE-2) scores [13]. This is in stark contrast to the situation of anaesthesia of an otherwise well patient undergoing surgery in an operating room where the air exchange rate is a minimum of 20/hour, and in some circumstances may be as high as 90/hour, being treated as ‘at risk’ of SARS-CoV-2 infection as a precautionary measure. Designation as an AGP has led to the procedure overshadowing the context in which it is being performed.

The healthcare system was also heavily impacted by the consequences of an AGP increasing the risk of furlough for a HCW. Inadvertent contact with a COVID-positive patient during an AGP increased the risk of HCW being furloughed [14]. The consequent leave of absence reduced the available workforce available to the health facility, which translated into staffing challenges that led to reduced capacity for patient care.

## Recent key learnings with regards to AGPs

Emerging evidence into aerosol generation indicates that many procedures designated as AGPs at the beginning of the SARS-CoV-2 pandemic are not aerosol-generating. Aerosol generation requires occurrence of shear forces due to high velocity gas flow across a gas–liquid interface [15]. Many procedures designated as AGPs do not inherently produce these high shear forces and are therefore unlikely to result in true aerosol generation. As such, the relevance of AGPs to transmission of SARS-CoV-2 has recently been questioned [15–21].

### Intubation and extubation

An AGP has been defined as one in which the aerosol generated being greater than a cough [22].

The evidence for intubation and extubation being a risk factor for contracting an acute respiratory infection was articulated in a review by Tran et al. [2]. This review included as evidence five case–control and five retrospective cohort studies [2]. This was in the context of the SARS -1 epidemic. They described an increased risk of transmission in a variety of procedures, including intubation, non-invasive ventilation, tracheostomy and manual ventilation prior to intubation. This review did not present a scientific analysis of respiratory parameters and transmission. The clinical context in which the intubations occurred was not considered. The individual studies which formed the basis of this systematic review were all assessed by the authors as being “very low” with regards to quality of evidence. The authors [2] made the point that tracheal intubation may require HCWs to be in close proximity to a patient’s airway for prolonged periods of time and the association of transmission of SARS-CoV-1 in this setting would be biologically plausible. But they also asserted that the procedure itself was only potentially capable of generating aerosols.

More recently, evidence has been presented objectively measuring aerosol generation in the context of intubation and extubation. Brown et al. [23] conducted a quantitative study of the aerosol generation of the intubation and extubation sequence within an ultra-clean ventilation operating room. The average concentration of particles recorded during the intubation period was 500-fold lower than the mean concentration recorded during volitional coughs, with a maximum concentration 22-fold lower. Extubation produced a mean concentration which was 35-fold lower than that seen during a volitional cough but 15-fold greater than that seen during intubation [23]. Brown et al. [23] findings supported the notion that the intubation sequence inclusive of face mask ventilation and extubation sequence is not an AGP.

Dhillon et al. [24], utilizing a different model of aerosol detection, identified that facemask ventilation, tracheal tube insertion and cuff inflation generated small particles 30–300 times above background noise that remained suspended in airflows and spread from the patient’s facial region throughout the confines of the operating room. The highest levels occurred with bag-and-mask ventilation prior to intubation. These findings seemingly contradict the findings of Brown [23]. This has led to an urgent call for consensus [17]. Dhillon [25] and Nestor [18] have provided commentary on the apparent disparity in results.

Nestor [18] elaborates that both of the studies demonstrated that the level of aerosols however was less on average than a volitional cough. The two studies were congruent in their findings that intubation and extubation produce fewer aerosols than a cough. Average volitional cough produced particles in excess of the average amount of aerosol generated by airway management in either study [18]. If a procedure is producing less than the amount of aerosols than a cough, then that procedure cannot and should not be classified as an AGP [22].

Importantly, neither of these studies [18, 24] measures virus particles nor infectivity. The clinical risk of respired respiratory pathogens being a function of emission rate, the size of distribution of particles carrying the pathogen and the removal rate constant [26]. Complex dynamics not considered in these studies.

### Supraglottic airways

Supraglottic airway insertion in the OR is commonly classified as an AGP. Yet supportive data for this classification have not been demonstrated. Shrimpton et al. [27] quantified the aerosol generation with supraglottic airway insertion and removal. Comparing this intervention to quiet tidal breathing and a volitional cough. They demonstrated average aerosol concentration detected during supraglottic airway insertion and removal was no different to tidal breathing. In addition, supraglottic airway insertion/removal sequences produced < 4% of the aerosol compared with a single cough [28]. Complicated insertion on one patient did lead to a measurable increase in aerosol production [28].

Objective data does not provide evidence to support the notion of uncomplicated supraglottic airway use being an AGP.

### Oxygen therapy and non-invasive ventilation

The provision of high flow oxygen and non-invasive positive pressure ventilation are important tools in the management of patients with respiratory failure. These modalities have also been classified as AGPs. In a healthy volunteer model, Gaeckle et al. [29] demonstrated that the provision of these therapies did not significantly increase the measured aerosol concentration with either therapy. This was consistent during normal breathing, talking, deep breathing and coughing. They concluded that aerosol generation was influenced more by the pattern of breathing and coughing than the mode of oxygen delivery.

Wilson et al. [30] examined three respiratory therapies; high flow nasal oxygen (HFNO), single and dual circuit non-invasive ventilation therapies, which are currently designated as AGP’s. They compared respiratory emissions in healthy volunteers during different respiratory activities. Activities such as talking, exercise, shouting, forced expirations and coughing increased measured emitted respiratory particles. They demonstrated that the respiratory therapies increased particle emissions relative to quiet breathing. However, when the therapies were in conjunction with exertional respiratory activities the measured respiratory emissions were less than the exertional respiratory therapies alone.

Hamilton et al. [31] measured aerosols with CPAP and HFNO utilising two methodologies. They demonstrated these modalities produced less aerosol than breathing, speaking, and coughing (even with large > 50 L/min face mask leaks). Coughing was associated with the highest aerosol emissions of any recorded activity. HFNO was associated with aerosol emission, however, this was from the machine. Generated particles were small, passing from the machine through the patient and to the detector without coalescence with respiratory aerosol, thereby unlikely to carry viral particles [31].

Gaeckle et al. [29] tested non-humidified nasal cannula, face mask, heated and humidified high-flow nasal cannula, and non-invasive positive-pressure ventilation. Aerosol generation was measured with each oxygen mode while participants performed manoeuvres of normal breathing, talking, deep breathing, and coughing. Cough significantly increased the number of particles measured. Measured aerosol concentration did not significantly increase with the use of either humidified high-flow nasal cannula or non-invasive positive-pressure ventilation. This was the case during normal breathing, talking, deep breathing, and coughing.

These respiratory therapies have an essential place in the treatment of respiratory failure however, collectively these studies provide data the interventions which have been designated as AGPs, do not produce aerosols.

### Other corroborating evidence

Several investigators have failed to prove the presence of viral particles in the air in association with AGPs. Thompson et al. [32] during the H1N1 influenza pandemic, concluded that the amount of H1N1 RNA in aerosols near patients having aerosol generating procedures, including intubation was not significantly increased. Recently, Conway et al. [33] detected airborne SARS-CoV-2 in in the atmosphere in both ward and ICU areas caring for COVID-19 patients. Airborne SARS-CoV-2 however was detected less frequently in the ICU area in comparison to the ward area. ICU being an environment within which, AGPs tend to occur to more frequently. The authors speculated that these patients being in the later stages of disease may have less viral replication and use of respiratory devices, which reduce aerosol generation.

## Future direction

The evidence that AGPs were a high-risk transmission event at the start of the current pandemic was suppositional and precautionary. A lack of understanding of the physiological airflow events during airway instrumentation processes led to a misguided conclusion with a motivation to protect HCWs.

We would join others [16] in suggesting that the concept of AGPs be abandoned in favour of a more nuanced approach to risk stratification for transmission of SARS-CoV-2, based on multiple factors. Individualised assessment would be based on risk factors consisting of patient symptoms (coughing, sneezing, tachypnoea), disease severity, distance from infected patient, ventilation in the environment, and duration [15]. Sustained proximity to a highly symptomatic patient in a poorly ventilated environment for a protracted period, encompasses the clinical context as the dominant risk. This *may* occur during airway management and intubation. But the concept of intubation and other airway interventions being an independent risk factor of infective risk, by virtue of being an AGP, is unsupported by contemporary evidence.

HCW protection should be based on a hierarchy of controls, with particular emphasis on viral removal through improved ventilation strategies, as well as protecting individual HCWs, through vaccination and appropriate use of PPE. Detailed consideration of a host of risk factors (Table 2) is more appropriate for instituting levels of infection control procedures.

**Table 2:** Risk factors for transmission of airborne pathogens

*Patient infectivity*
Nature of pathogen
Probability of infection
Time course of infection
*Infective airborne particle generation*
Cough
Tachypnoea
*Exposure*
Physical proximity to patient
Temporal proximity to patient
Duration of exposure
Ventilation
Personal protective equipment
*Practitioner vulnerability*
Age
Co-morbidities
Vaccination status

## Conclusion

The concept of AGPs being an independent risk of transmission through aerosolisation containing live virus and being a hazard to HCWs in particular, was extrapolated from prior pandemic experiences. With time, we have developed a better understanding of SARS-CoV-2 and the influence of AGPs on the transmission of this disease. Evidence has emerged that casts significant doubt on aerosol generation during airway procedures.

Coincidentally, the clinical practice guidelines during the COVID-19 pandemic advocating the use of airborne PPE kept HCWs involved in airway management safe and prevented HCW infections. However, the focus on individual procedures as “aerosol generating” that required extra time-consuming measures (such as OR clearance times) or those that should be avoided (such as HFNO_2_) may have been misplaced. Although these procedures may still be considered as “at risk” procedures if they pose an increased risk of infection to staff through prolonged and close exposure to an infectious patient.

The term “aerosol generating procedure” should be abandoned as an independent risk factor for the transmission of SARS-CoV-2. Instead, a reemphasis upon the clinical context of exposure should be utilized to guide infection control precautions.

## Data Availability

Not applicable, no data collected.
